# Empagliflozin Limits Myocardial Infarction *in Vivo* and Cell Death *in Vitro*: Role of STAT3, Mitochondria, and Redox Aspects

**DOI:** 10.3389/fphys.2017.01077

**Published:** 2017-12-19

**Authors:** Ioanna Andreadou, Panagiotis Efentakis, Evangelos Balafas, Gabriele Togliatto, Constantinos H. Davos, Aimilia Varela, Constantinos A. Dimitriou, Panagiota-Efstathia Nikolaou, Eirini Maratou, Vaia Lambadiari, Ignatios Ikonomidis, Nikolaos Kostomitsopoulos, Maria F. Brizzi, George Dimitriadis, Efstathios K. Iliodromitis

**Affiliations:** ^1^Laboratory of Pharmacology, Faculty of Pharmacy, School of Health Sciences, National and Kapodistrian University of Athens, Athens, Greece; ^2^Academy of Athens Biomedical Research Foundation, Centre of Clinical Experimental Surgery and Translational Research, Athens, Greece; ^3^Department of Medical Sciences, University of Turin, Turin, Italy; ^4^Cardiovascular Research Laboratory, Biomedical Research Foundation, Academy of Athens, Athens, Greece; ^5^Hellenic National Center for Research, Prevention and Treatment of Diabetes Mellitus and Its Complications, Athens, Greece; ^6^2nd Department of Internal Medicine, Research Institute and Diabetes Center, National and Kapodistrian University of Athens, “Attikon” University Hospital, Athens, Greece; ^7^2nd University Department of Cardiology, Medical School, National and Kapodistrian University of Athens, Athens, Greece

**Keywords:** empagliflozin, cardioprotection, infarct size, cardiac function, molecular signaling, STAT3 pathway

## Abstract

Empagliflozin (EMPA), a drug approved for type 2 diabetes management, reduced cardiovascular death but is unknown if it reduces myocardial infarction. We sought to investigate: (i) the effect of EMPA on myocardial function and infarct size after ischemia/reperfusion in mice fed with western diet (WD), (ii) the underlying signaling pathways, (iii) its effects on cell survival in rat embryonic-heart-derived cardiomyoblasts (H9C2) and endothelial cells (ECs). To facilitate the aforementioned aims, mice were initially randomized in Control and EMPA groups and were subjected to 30 min ischemia and 2 h reperfusion. EMPA reduced body weight, blood glucose levels, and mean arterial pressure. Cholesterol, triglyceride, and AGEs remained unchanged. Left ventricular fractional shortening was improved (43.97 ± 0.92 vs. 40.75 ± 0.61%) and infarct size reduced (33.2 ± 0.01 vs. 17.6 ± 0.02%). In a second series of experiments, mice were subjected to the above interventions up to the 10th min of reperfusion and myocardial biopsies were obtained for assessment of the signaling cascade. STAT3 was increased in parallel with reduced levels of malondialdehyde (MDA) and reduced expression of myocardial iNOS and interleukin-6. Cell viability and ATP content were increased in H9C2 and in ECs. While, STAT3 phosphorylation is known to bestow infarct sparing properties through interaction with mitochondria, we observed that EMPA did not directly alter the mitochondrial calcium retention capacity (CRC); therefore, its effect in reducing myocardial infarction is STAT3 dependent. In conclusion, EMPA improves myocardial function and reduces infarct size as well as improves redox regulation by decreasing iNOS expression and subsequently lipid peroxidation as shown by its surrogate marker MDA. The mechanisms of action implicate the activation of STAT3 anti-oxidant and anti-inflammatory properties.

## Introduction

In the EMPA-REG OUTCOME trial, empagliflozin (EMPA), a selective inhibitor of the sodium glucose co-transporter 2 (SGLT2), reduced the risk of the combined endpoint of hospitalization for heart failure or cardiovascular death, in type 2 diabetic patients (T2D) (Fitchett et al., 2016). The mechanism by which EMPA induces cardiovascular benefits is obscure. However, the discrepancy in the Hazards ratios (HRs) for non-fatal myocardial infarctions (HR 0.87 [95% CI 0.70, 1.09]) and non-fatal strokes (HR 1.24 [0.92, 1.67]) indicates that benefits of EMPA may do not involve classical effects on atherosclerosis (Vettor et al., 2017). Although the results of the EMPA-REG trial were impressive, the cardioprotective mechanisms of the drug are still speculative. There is no *in vitro*, or *in vivo* study investigating if EMPA exerts its cardioprotection through the reduction of myocardial infarction after ischemia/reperfusion (I/R) injury. In the present study we investigated the effect of EMPA on myocardial function and infarction after I/R, in a mouse model with diet-induced T2D. In order to achieve this goal we recruited a murine model of diet induced metabolic syndrome, known to manifest T2D. The C57BL/6 mouse strain has been indicated to be prone in T2D and atherosclerosis following a high-fat, WD for prolonged time (Surwit et al., 1988; Phillips et al., 2003). We selected a dose of 10 mg/kg/day of EMPA which has been calculated based on the inter-species pharmacokinetic and metabolic parameters between rodents and humans (e.g., half-life ~1–2 h in rodent and 10–12 h in man). This dose has been previously shown to correspond to the equivalent active dose in humans (Cheng et al., 2016). Additionally, since we know that SGLT2 is highly specifically expressed in the kidney and very minimally in the heart (Chen et al., 2017) we sought to investigate the mechanism by which EMPA may exert cardioprotective effects based on intracellular signaling cascades independent of SGLT2, as it has been shown that EMPA exerts pleiotropic effects in aorta and in adipose tissue (Steven et al., 2017).

To further investigate a direct cardioprotective effect of EMPA, we evaluated its effect on an *in-vitro* model of injury: we exposed rat embryonic-heart-derived cardiomyoblasts (H9C2) and endothelial cells (ECs) to hypoxia/reoxygenation. Moreover, taking under consideration that mitochondria and redox aspects play very important role in cardioprotection (Penna et al., 2013; Pagliaro and Penna, 2015) we also focused on redox signaling and mitochondrial susceptibility to transition.

## Materials and methods

For Complete Methods please see Supplementary Material.

### *In Vivo* experiments

#### Animals

A total of thirty five male 8-week old C57BL/6J animals were treated according to the Directive 2010/63/UE European Convention for the Protection of Vertebrate Animals used for Experimental and other Scientific Purposes, and conformed to the Guide for the Care and Use of Laboratory Animals published by the US National Institutes of Health (NIH Publication No. 85-23, revised 1985). The experimental protocol used in this study was approved by Ethical Committee of University of Athens and the Veterinary Authorities of Region of Athens Greece (License Number: 1758/24/3/2017).

#### Experimental protocol

Mice were fed a Western diet (WD) (TD 88137, Harlan-Teklad; containing 21% fat by weight, 0.15% by weight cholesterol, and 19.5% by weight casein without sodium cholate), for 14 weeks (Surwit et al., 1988; Phillips et al., 2003). At the 8th week of feeding mice were randomized into two groups: (i) Control group (*n* = 15) in which DMSO 5% in Water For Injection (WFI) solution was given through gavage for additional 6 weeks and (ii) EMPA group (*n* = 15), in which EMPA was administered (10 mg/kg/day) in 5% DMSO/WFI through gavage for additional 6 weeks.

The EMPA dose was selected according to previous reports (Oelze et al., 2014; Habibi et al., 2017). At the end of treatment eighteen mice (*n* = 9 per group) subjected to 30 min myocardial ischemia followed by 2h reperfusion to determine the infarct size. Twelve mice (*n* = 6 per group) were subjected to the above interventions up to the 10th min of reperfusion, to obtain myocardial biopsies from the ischemic area for Western Blot analysis.

The following parameters were determined at baseline (Day 0), at the end of 8th week and at the end of the 14th week (end of treatment): body weight (BW), glucose levels. The following parameters were determined at baseline (Day 0), and at the end of the 14th week (end of treatment): cholesterol and triglycerides, arterial pressure, and myocardial function by echocardiography. Malondialdehyde (MDA) as a marker of lipid peroxidation was determined at the end of 8th week and at the end of 14th week, while advanced glycation end products (AGE) were determined at the end of 14th week.

#### Murine model of ischemia-reperfusion injury

##### Anesthesia/surgical preparation

General anesthesia was induced *via* intraperitoneal injection of a mixture containing ketamine, xylazine, and atropine (100, 20, and 0.6 mg/kg respectively). A cuffed tracheal tube was placed via a tracheotomy (ventral midline incision) for mechanical ventilation of the lungs with a 95% O_2_ via a volume-cycled animal ventilator (150 strokes/min, tidal volume 200 μl). Consequently, the animals were placed in left-lateral recumbence and the chest was surgically opened with a left thoracotomy. The chest was opened through the left fourth intercostal space. The beating heart was exposed and the pericardium was incised. The left anterior descending coronary artery was ligated with a 6-0 silk suture. The occlusion of coronary artery in this region leads to ischemia of a large area of the anterolateral and apical left ventricular wall. Ischemia was induced by tightening of the suture against a small piece of polyethylene tubing. Ischemia resulted in a change in the color (i.e., cyanotic) of the myocardium and was maintained for 30 min. At the end of the ischemic period, the suture was released in order to induce reperfusion of the myocardium for up to 2 h. In order to determine/quantify the degree of irreversible myocardial injury (i.e., infarction) resulting from the ischemia and reperfusion insult with and without indicated treatment, infarct areas were evaluated. For this reason, myocardium was excised and aorta was catheterized. After the tightening of the suture, Evan's Blue solution (2.5% in Water for Injection-WFI) was infused through the catheter for the identification of the normally perfused part of the myocardium. Afterwards, hearts were frozen for 24 h and then sliced in 1–2 mm sections, which were incubated for 20 min in Triphenyl-Tetrazolium Chloride Solution (TTC, 1% in PBS pH = 7.4) for the identification of the area-at-risk and the infarct area (Bibli et al., 2015).

#### Histopathology/histomorphometery

For each heart, the overall size of the myocardial slice (All/A), the area-at-risk (Risk/R) and the infarct (Infarct/I) were determined. R was expressed as a percentage of the A area (R/A %), and I was expressed as a percentage of the R (I/R %) (Bibli et al., 2015).

#### Blood biochemistry

Mice were fasted overnight prior to blood sampling. Blood was collected from the tail vein by puncturing the vessel vertically with a 23-gauge needle at baseline, at the 8th week and at the end of the treatment periods. Blood glucose levels were measured by using a hand-held glucometer (Onetouch Verio IQ Lifescan, Johnson & Johnson Company).

For the assessment of lipid levels and MDA measurement, blood samples were collected at the beginning of the treatment period and at the end of the study. Blood was centrifuged and plasma was stored at −80°C. Plasma cholesterol and triglyceride concentrations were measured spectrophotometrically using commercial kits (DiaSys Diagnostic Systems GmbH, Cholesterol FS 10130021, and Triglycerides FS 10571 021). The MDA concentration was determined spectrophotometrically at 586 nm and expressed as μM (Oxford Biomedical Research Colorimetric Assay for lipid peroxidation) as we have previously described (Andreadou et al., 2004).

#### Arterial pressure (AP) monitoring

At baseline and at the end of treatment, non-invasive arterial pressure was measured on awake mice using CODA Monitor tail-cuff system (Kent Scientific Co, Torrington, CT USA). The mice were placed for 10 min inside a heated chamber (34°C) and then positioned in a restrainer over a heating pad. They were allowed a 5 min acclimation period following cuff positioning and 20 consecutive AP determination cycles we conducted. All measurements were recorded on the CODA software and presented in Table 1. In case signs of discomfort were present, the animal was returned to the heated chamber and re-examined 1 h later.

**Table 1:** Cholesterol and triglyceride levels, mean arterial pressure values and echocardiography data at baseline and after 14 weeks of treatment in Control (*n* = 10) and EMPA (*n* = 10) groups.

	**Control**	**EMPA**
Triglycerides Baseline (mg/dl)	37.91 ± 3.28	54.36 ± 10.12
Triglycerides 14 weeks (mg/dl)	36.93 ± 9.05^*^	55.41 ± 8.59^*^
Cholesterol Baseline (mg/dl)	145.5 ± 16	139.2 ± 12
Cholesterol 14 weeks (mg/dl)	233.6 ± 43^*^	191.9 ± 20.92^*^
Diastolic AP Baseline (mmHg)	80.51 ± 2.0	79.17 ± 2.2
Diastolic AP 14 weeks (mmHg)	73.18 ± 2.1	70.87 ± 2.1^*^
Systolic AP Baseline (mmHg)	106.70 ± 2.3	105.35 ± 2.4
Systolic AP 14 weeks (mmHg)	102.55 ± 2.0	96.91 ± 2.2^*^
Mean Arterial Pressure Baseline (mmHg)	88.9 ± 2.32	87.56 ± 2.4
Mean Arterial Pressure 14 weeks (mmHg)	82.58 ± 2.6	79.22 ± 2.1^*^
HR Baseline	592.20 ± 5.27	627.90 ± 11.29^#^
HR 14 weeks	702.37 ± 10.31^*^	687.20 ± 5.46^*^
**ECHOCARDIOGRAPHY**		
LVEDD, mm Baseline	2.90 ± 0.07	2.96 ± 0.06
LVEDD, mm 14 weeks	3.20 ± 0.07^*^	3.04 ± 0.07
LVESD, mm Baseline	1.54 ± 0.05	1.59 ± 0.03
LVESD, mm 14 weeks	1.90 ± 0.05^*^	1.71 ± 0.06^#^
PWd, mm Baseline	0.79 ± 0.01	0.77 ± 0.01
PWd, mm 14 weeks	0.74 ± 0.01^*^	0.76 ± 0.01^#^
PWs, mm Baseline	1.27 ± 0.01	1.27 ± 0.01
PWs, mm 14 weeks	1.23 ± 0.01^*^	1.24 ± 0.01^*^
FS% Baseline	47.07 ± 0.65	46.14 ± 0.31
FS% 14 weeks	40.75 ± 0.61^*^	43.97 ± 0.92^#^
r/h Baseline	1.84 ± 0.06	1.91 ± 0.05
r/h 14 weeks	2.15 ± 0.07^*^	1.98 ± 0.05

* *p < 0.05 vs. Baseline*,

# *p < 0.05 vs. Control group. Left ventricular (LV) end-diastolic (EDD) and end-systolic dimension (ESD); posterior wall thickness (PW) in diastole (d) and systole (s); percentage fractional shortening (FS%); ratio of LV radius to PWT (r/h)*.

#### Echocardiography

At baseline and at the end of the treatment, M-mode echocardiography was performed as previously described (Papathanasiou et al., 2015) to measure left ventricular (LV) end-diastolic diameter (EDD), LV end-systolic diameter (ESD), and LV posterior wall thickness at diastole (PWT) and to calculate the ratio of LV radius to PWT (r/h) and the percentage of LV fractional shortening FS (%).

#### Western blot analysis in myocardial tissue

Myocardial tissues were pulverized and the powder was homogenized in lysis buffer (1% Triton X-100, 20 mM Tris pH 7.4–7.6, 150 mM NaCl, 50 mM NaF, 1 mM EDTA, 1 mM EGTA, 1 mM Glycerolphosphatase, 1% SDS, 100 mM PMSF, and 0.1% protease phosphatase inhibitor cocktail). After centrifugation at 11,000 g for 15 min at 4°C, supernatants were collected and protein content was assessed using the Lowry method. The supernatant was mixed with Dave's buffer (4% SDS, 10% 2-mercaptoethanol, 20% glycerol, 0.004% bromophenyl blue, and 0.125 M Tris·HCl). The samples were boiled at 100°C for 10 min and stored at −80°C. An equal amount of protein was loaded in each well and then separated by sodium dodecylsulfate-polyacrylamide gel electrophoresis 7.5–11% and transferred onto a polyvinylidene difluoride membrane (PVDF). After blocking with 5% non-fat dry milk, membranes were incubated overnight at 4°C with the following primary antibodies:

p-eNOS (Ser1177), eNOS, p-Akt (Ser473), Akt, p-ERK 1/2 (Thr202/Tyr204), p-GSK3β (Ser9), t-GSK3β, pAMPKα(Ser172), tAMPKα, p-STAT3 (Tyr705), t-STAT3, iNOS, p-NF-κB (p65) (Ser536), t- NF-κB (p65), GAPDH, β-tubulin, β-actin (Cell Signaling Technology, Beverly, MA, USA) and IL-6 (Santa-Cruz Biotechnology Inc., USA). PVDF membranes were then incubated with secondary antibodies for 2 h at room temperature (goat anti-mouse and goat anti-rabbit HRP; Cell Signaling Technology, Beverly, MA, USA) and developed using the GE Healthcare ECL Western Blotting Detection Reagents (Thermo Scientific Technologies, Bioanalytica, Athens, Greece). Relative densitometry was determined using a computerized software package (NIH, USA), and relative ratios were used for statistical analysis (Bibli et al., 2015, 2016).

#### Advanced glycation end-products (AGE) fluorescence measurement

The fluorescence intensity of plasma samples was measured at 440 nm after excitation at 370 nm, using a fluorescence spectrophotometer (Infinite® 200 PRO NanoQuant Plate Reader) operating at room temperature. Emission and excitation slit widths were set at 5 nm. Fluorescence was expressed as the Intergrated Fluorescence intensity in arbitrary units (AU), as previously described (Yanagisawa et al., 1998).

### *In Vitro* experiments

#### Effects on cellular level

##### Cell culture and in vitro hypoxia/reoxygenation assay

Endothelial cells (ECs) were purchased from Lonza (Basel, Switzerland) and cultured as described by the manufacturer's instructions. The embryonic rat heart-derived cell line H9C2 was obtained from American Type Culture Collection (Manassas, VA, USA). Cells were maintained in DMEM high glucose (HG) with 10% FBS, 4 mM glutamine and grown to subconfluence prior to experiments. In parallel experiments H9C2 cells were cultured for 24 h either alone or in combination with 1 mg/ml AGE and untreated or treated with EMPA (100 or 500 nM) (Panchapakesan et al., 2013). ECs, maintained for 24 h in 5% FBS, were cultured either alone or in combination with AGE and untreated or treated with EMPA (100 or 500 nM). At day 2, cells were subjected to *in vitro* hypoxia for 24 h (5% CO_2_/95% N_2_ humidified atmosphere, yielding 1% O_2_ concentrations) and subsequently reoxygenated for 3 h (75% N_2_, 20% O_2_, and 5% CO_2_).

##### ATP content assay

To determine the level of cellular ATP content as an indirect measurement of viable cells, ATP content was performed using ATP assay kit according to manufacturer's instructions (Sigma Aldrich). Briefly, ECs and H9C2, treated as indicated, were subjected to *in vitro* ischemia for 24 h. After reoxygenation, ATP was quantitatively determined by measuring luminescence generated in an ATP-dependent luciferin-luciferase bioluminescence assay. A standard curve was used in each experiment, and the samples were diluted to be in the linear range of the standard curve. All experiments were performed in triplicate.

##### Cell viability assay

Cell viability was assessed by MTT as described previously (Baldanzi et al., 2002). Cells were seeded on 96-well plates at 5 × 10^3^ cells/well. ECs and H9C2 which had either been treated with the indicated stimuli or had been left untreated were subjected to *in vitro* ischemia and reoxygenation. After treatments, cells were incubated with 1 mg/ml MTT for 2 h. Subsequently, the medium was removed and 100 μl of dimethylsulfoxide (DMSO) was added to each well. After 1 h, the 96-well plate was read by an enzyme-linked immunosorbent assay (ELISA) reader at 570 nm for absorbance density values to determine cell viability. All experiments were performed in triplicate.

##### Western blot analysis in cells

Cells were lysed (50 mM Tris HCl [pH 8.3], 1% Triton X-100, 10 mM PMSF, 100 U/mL aprotinin, 10 μM/mL leupeptin) and protein concentrations were obtained as previously described (Togliatto et al., 2011). Proteins (50 μg) were subjected to SDS-PAGE, transferred into nitrocellulose membrane, blotted with the indicated antibodies (anti-RAGE antibody cat. No. sc-8230, and anti-b-actin antibody cat. No. sc-47778, purchased from Santa Cruz, Biotechnology, Germany) and processed as previously described (Togliatto et al., 2011). Densitometric analysis was used to calculate the differences in the fold induction of protein level normalized to β-actin. Values are reported as relative amounts.

#### Effects on subcellular level

##### Mitochondrial isolation

Five additional C57BL/6 mice weighting 25–30 g were euthanized by cervical dislocation, and their hearts were quickly excised, rinsed, and cut in the isolation buffer (225 mM mannitol, 75 mM sucrose, 10 mM HEPES-Tris, 1 mM EGTA-Tris, pH 7.4). Then, the tissue was homogenized in the isolation buffer supplemented with 0.1 mg/ml Nagarse by using a glass-Teflon homogenizer. The homogenate was diluted in isolation buffer supplemented with 0.2% w/v bovine serum albumin, centrifuged at 500 g at 4°C, and filtered through a 150-mm mesh for the removal of cellular debris. The supernatant was further centrifuged at 8,000 g to separate the mitochondrial from the cytosolic fraction. The pellet, consisting of mitochondria was washed with isolation buffer without bovine serum albumin and centrifuged at 8,000 g. The final pellet was used for protein determination and further assays.

##### Calcium retention capacity assay

The calcium retention capacity (CRC) assay was performed as previously described (Chatzianastasiou et al., 2016) in order to determine the susceptibility of mitochondria to undergo permeability transition. Isolated mitochondria were diluted in mitochondrial assay buffer (KCl 137 mM, KH2PO4 2 mM, HEPES 20 mM, EGTA 20 mM, glutamate/malate 5 mM, pH 7.2) at a concentration of 0.25 mg/ml. Extramitochondrial Ca^2+^ was measured by Calcium Green-5N (1 mM) fluorescence using a Fluoroskan Ascent FL plate reader (Thermo Electron, Waltham, MA). Each minute, pulses of 10 mM Ca^2+^ were added to each well, up to a point when the accumulated Ca^2+^ was released due to mitochondrial transition through the opening of the mitochondrial permeability transition pore (PTP). Cyclosporine A (CsA) (1 mg/ml), was used as a positive control. Mitochondria were exposed to different concentrations of EMPA, and their CRC was determined: All experiments performed in five repetitions.

##### Statistical analysis

All values were denoted as means ± S.E.M. For animal studies and tissue experiments comparisons of numeric variables among the groups were analyzed using unpaired two-tailed Student's *t*-test, while those originating by different time points belonging to the same group were analyzed using paired two-tailed Student's *t*-test. A calculated *p* < 0.05 was considered to be statistically significant.

For mitochondria and cell culture experiments, the D'Agostino–Pearson test was used to test normality. Data on cell viability assay, ATP content assay and on densitometric analysis for Western blots were analyzed using 1-way ANOVA, followed by Tukey's multiple comparison test. The cut-off for statistical significance was set at *p* < 0.05 (^*^p < 0.05, ^**^p < 0.01, ^***^p < 0.001). All statistical analyses were carried out using GraphPad Prism version 5.04 (Graph Pad Software, Inc.).

## Results

### EMPA reduces body weight, mean arterial pressure, glucose, and lipid peroxidation levels without altering lipid levels and age products

**Figure 1:**
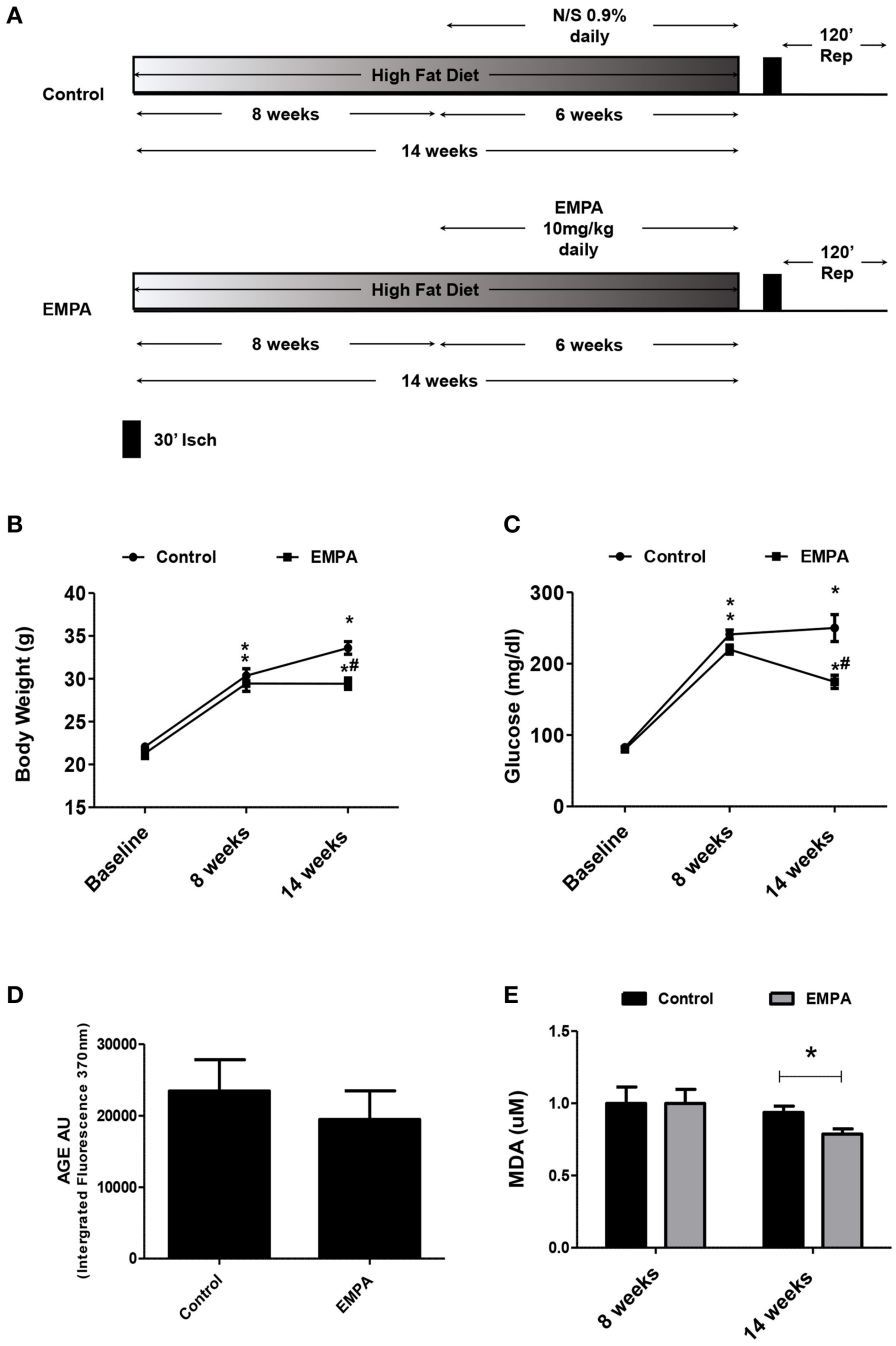
Empagliflozin reduces body weight, glucose levels, and lipid peroxidation levels without altering AGE products. **(A)** Experimental work flow. **(B)** Effects of diet manipulation and empagliflozin treatment on mice BW (^*^p < 0.05 vs. Baseline, ^#^*p* < 0.05 vs. Control). **(C)** Effects of diet manipulation and empagliflozin treatment on fasting glucose levels (^*^p < 0.05 vs. Baseline, ^#^*p* < 0.05 vs. Control). **(D)** AGE (AU) measured as Intergrated Fluorescence at emission 370 nm and **(E)** effects of diet manipulation and empagliflozin treatment on circulating MDA (uM) levels (^*^p < 0.05 vs. Control).

The experimental protocol of the study is illustrated in Figure 1A. In both groups, BW increased after 14 weeks of WD feeding. At the end of treatment, EMPA reduced significantly the BW (Figure 1B) and fast blood glucose levels (250 ± 18 mg/dL vs. 172 ± 8 mg/dL, ^*^p < 0.05, Figure 1C) in comparison to Control. Mean arterial pressure (MAP) was significantly reduced from baseline after 6 weeks treatment in the EMPA group (Table 1). Cholesterol and triglyceride levels did not differ between study groups at the end of treatment; however, they were significantly elevated compared to baseline values (Table 1). AGE levels were similar between the study groups at the end of treatment period (Figure 1D). Reduced levels of the biomarker of lipid peroxidation, malondialdehyde (MDA) in circulation was observed in the EMPA group compared to the Control group (Figure 1E, *p* < 0.05).

### Cardiac function is improved with EMPA pretreatment in mice treated with WD

Echocardiography revealed a reduction in the myocardial function after 14 weeks WD feeding as shown by the left ventricular fractional shortening (LVFS%; 43.97 ± 0.92 vs. 40.75 ± 0.61%, *p* = 0.001), due to deterioration of both LV end-systolic and end-diastolic dimensions (Table 1). These changes were not evident after EMPA treatment (Table 1, Figure S2).

### EMPA pretreatment reduces myocardial infarct size in mice treated with WD

**Figure 2:**
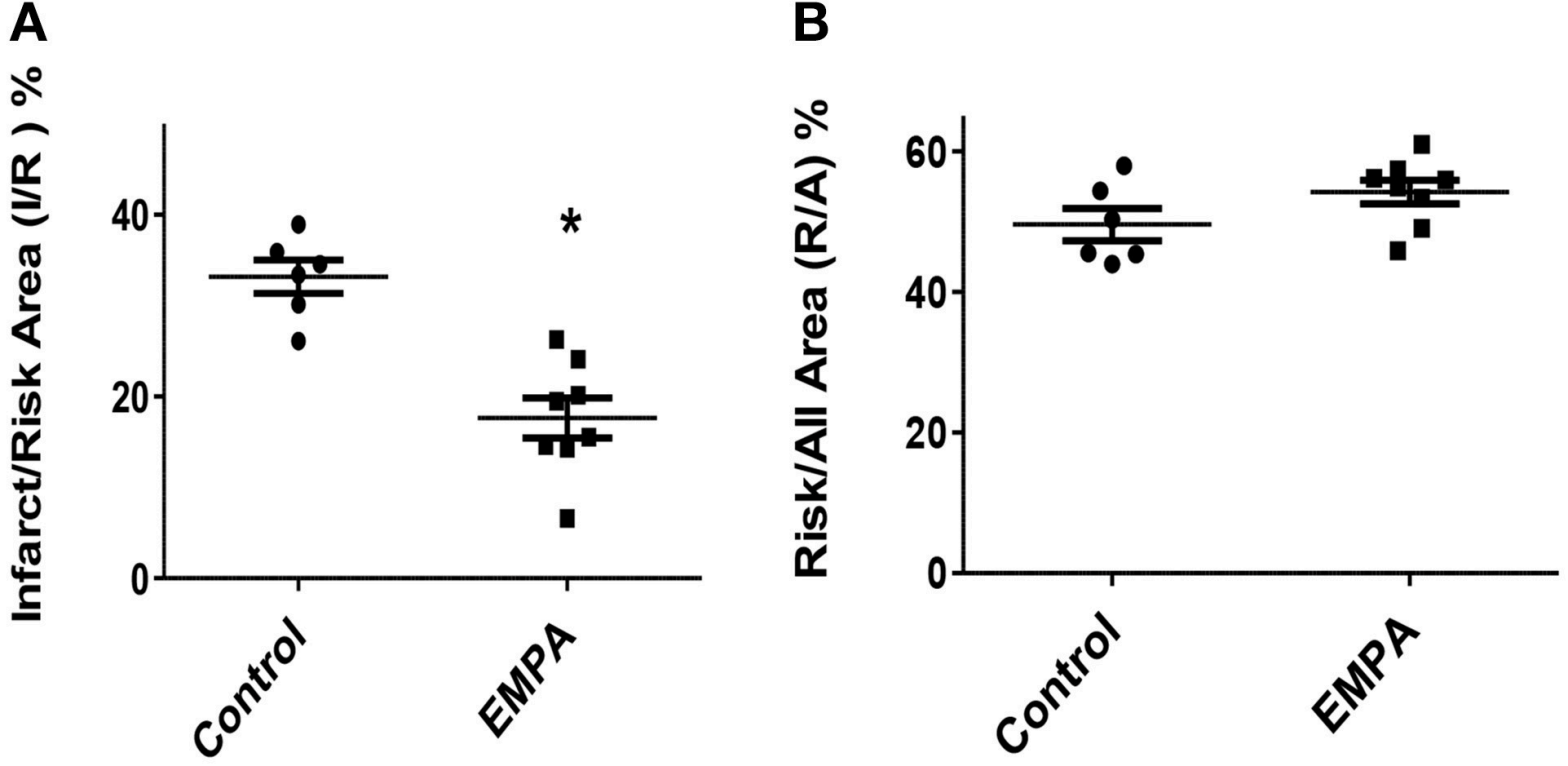
Empagliflozin pretreatment reduces myocardial infarct size in mice treated with WD. **(A)** Representative Graphs of Infarct/Risk Area (I/R) %. Individual animals represented as a scatter plot with dots in the Control group and squares in the EMPA group. Results plotted as Mean ± SEM (^*^p < 0.05 vs. Control). (**B)** Risk/All Area (R/A) %.

Three animals from the Control group and one animal from the EMPA group were excluded for different technical and/or hemodynamic reasons (severe bleeding and infarction detected outside the area at risk). As a result, 14 animals completed the study, Control (*n* = 6) EMPA (*n* = 8). Myocardial infarct size was reduced in EMPA group (33.2 ± 0.01 vs. 17.6 ± 0.02%, *p* < 0.05) (Figure 2A), with no difference in the area at risk (Figure 2B).

### EMPA pretreatment induces cardioprotection through STAT3 activation, independently of the reperfusion injury salvage kinase (RISK) pathway and AMPKα activation

**Figure 3:**
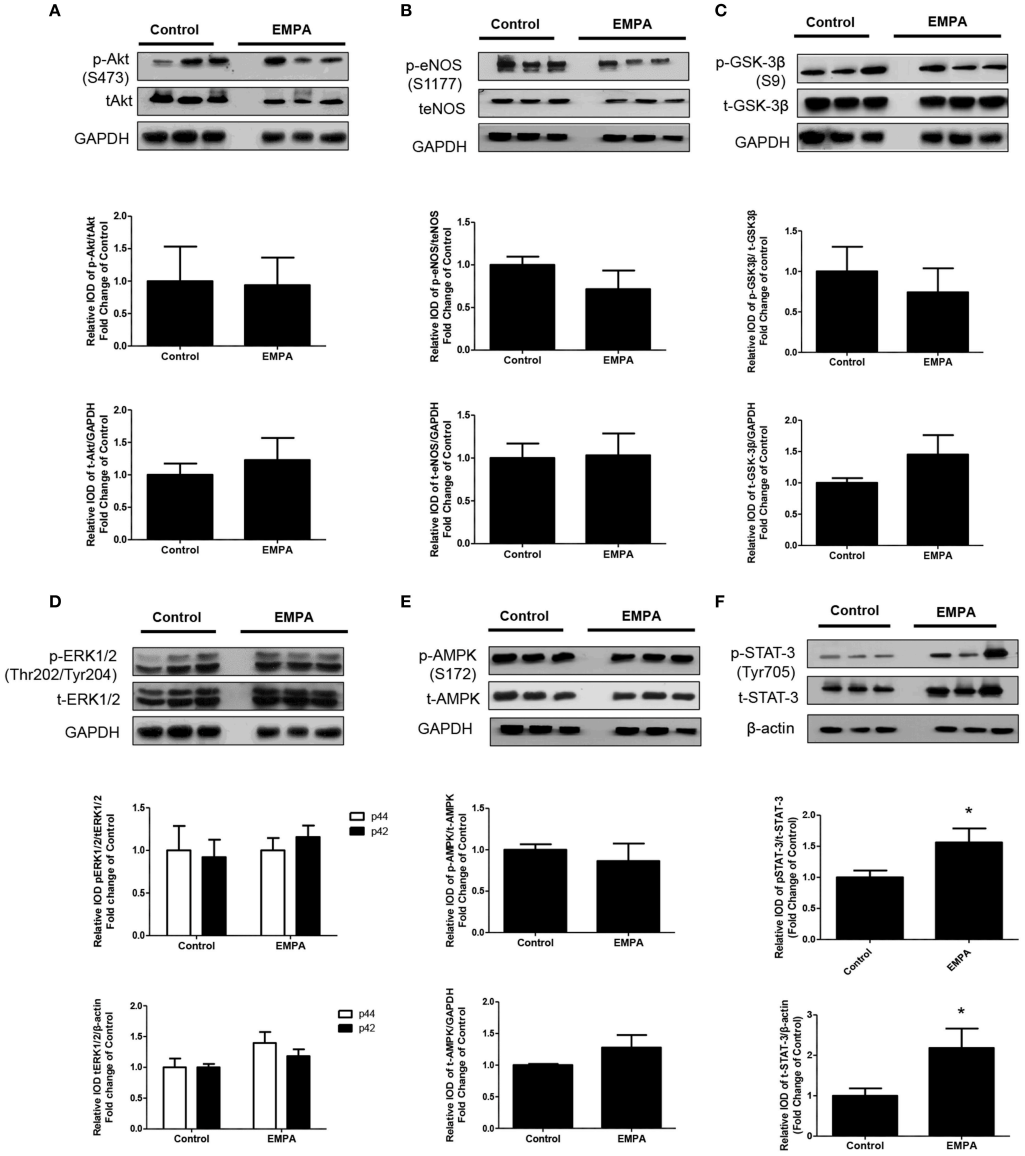
Empagliflozin induces cardioprotection through activation of STAT-3 and independently of RISK pathway and of AMPK activation. Representative western blots and relative densitometry graphs of **(A)** p-Akt (S473)/t-Akt and t-Akt/GAPDH **(B)** p-eNOS(S1177)/t-eNOS and t-eNOS/GAPDH **(C)** p-GSK-3β(S9)/t-GSK-3β and t-GSK-3β/GAPDH **(D)** p-ERK1/2 (Thr202/Tyr204)/t-ERK1/2 and t-ERK1/2/GAPDH **(E)** p-AMPKα(S172)/t-AMPKα and t-AMPKα/GAPDH **(F)** p-STAT3(Tyr705)/t-STAT3 and t-STAT3/β-actin (^*^p < 0.05 vs. Control). p-Akt, t-Akt **(A)** and p-ERK1/2, t-ERK1/2 **(D)** proteins were detected on the same gel and share the same image of GAPDH, serving as loading Control. This was also the case for p-eNOS, t-eNOS **(B)**, p-AMPKα, and t-AMPKα **(E)**.

**Figure 4:**
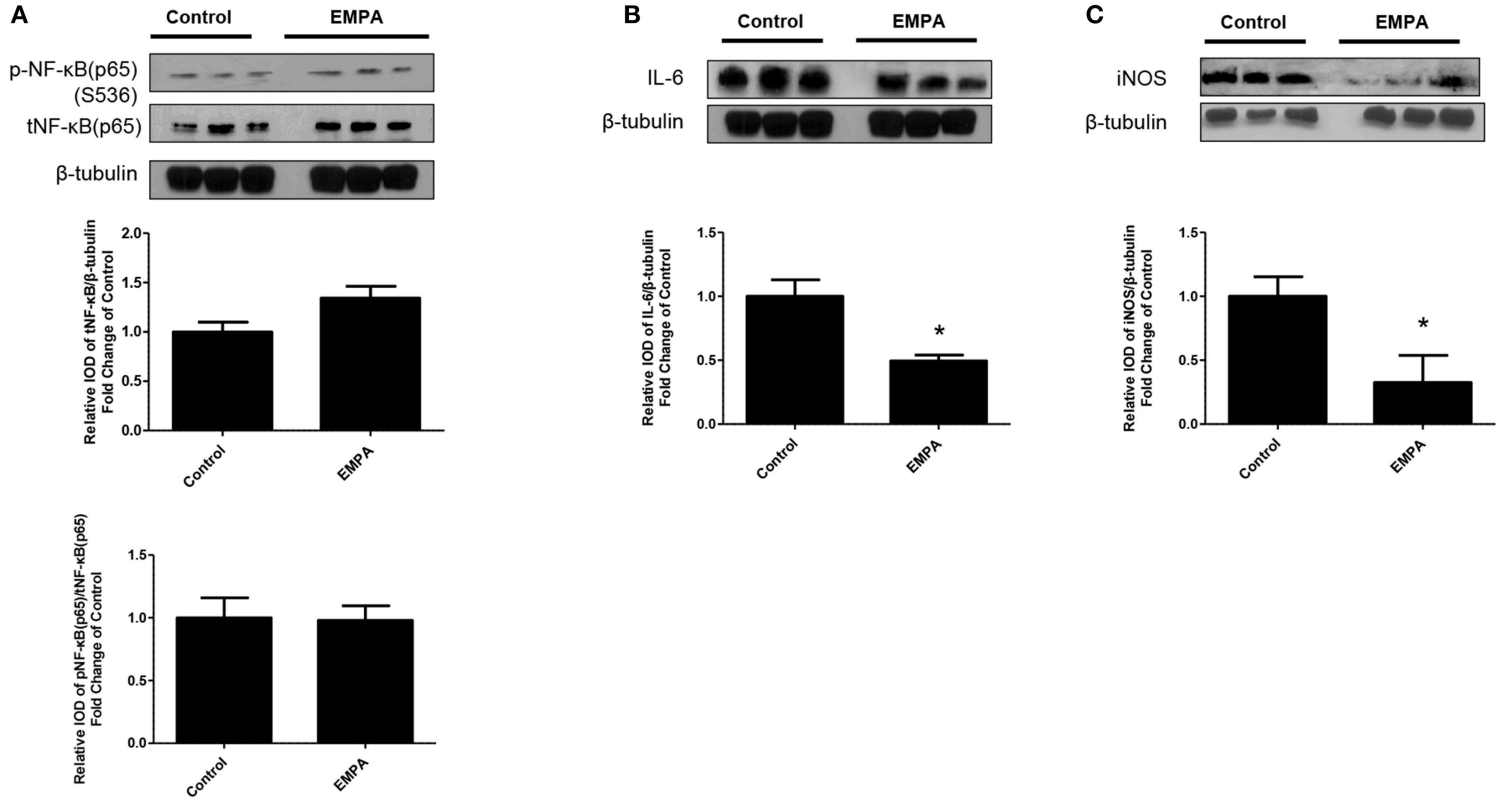
Empagliflozin reduces myocardial iNOS and IL-6 expression. Representative western blots and relative densitometry graphs of **(A)** p-NF-κB (p65) (S536)/ t-NF-κB (p65) and t-NF-κB (p65)/β-tubulin **(B)** IL-6/ β-tubulin (^*^p < 0.05 vs. Control) and **(C)** iNOS/ β-tubulin (^*^p < 0.05 vs. Control). p-NFκB(p65), t-NFkB(p65) **(A)** and IL-6 **(B)** were detected on the same gel and share the same image of β tubulin, serving as loading Control.

EMPA pretreatment did not activate Akt (Figure 3A), eNOS (Figure 3B), did not phosphorylate GSK3β on its inhibitory site (Figure 3C), did not activate p-44/p-42(ERK 1/2) (Figure 3D), and had no effect on AMPKα phosphorylation (Figure 3E). EMPA increased both STAT3 expression and phosphorylation (Figure 3F, *p* < 0.05) compared to the untreated group. Additionally, EMPA did change neither the phosphorylation nor the expression of nuclear factor kappa-light-chain-enhancer of activated B cells (NF-κB) (Figure 4A). Pretreatment with EMPA resulted in reduced levels of myocardial inteleukin-6 (IL-6) (Figure 4B, *p* < 0.05) and of inducible nitric oxide synthase (iNOS) expression (Figure 4C, *p* < 0.05).

### Evaluation of the effects of EMPA on isolated heart mitochondria

**Figure 5:**
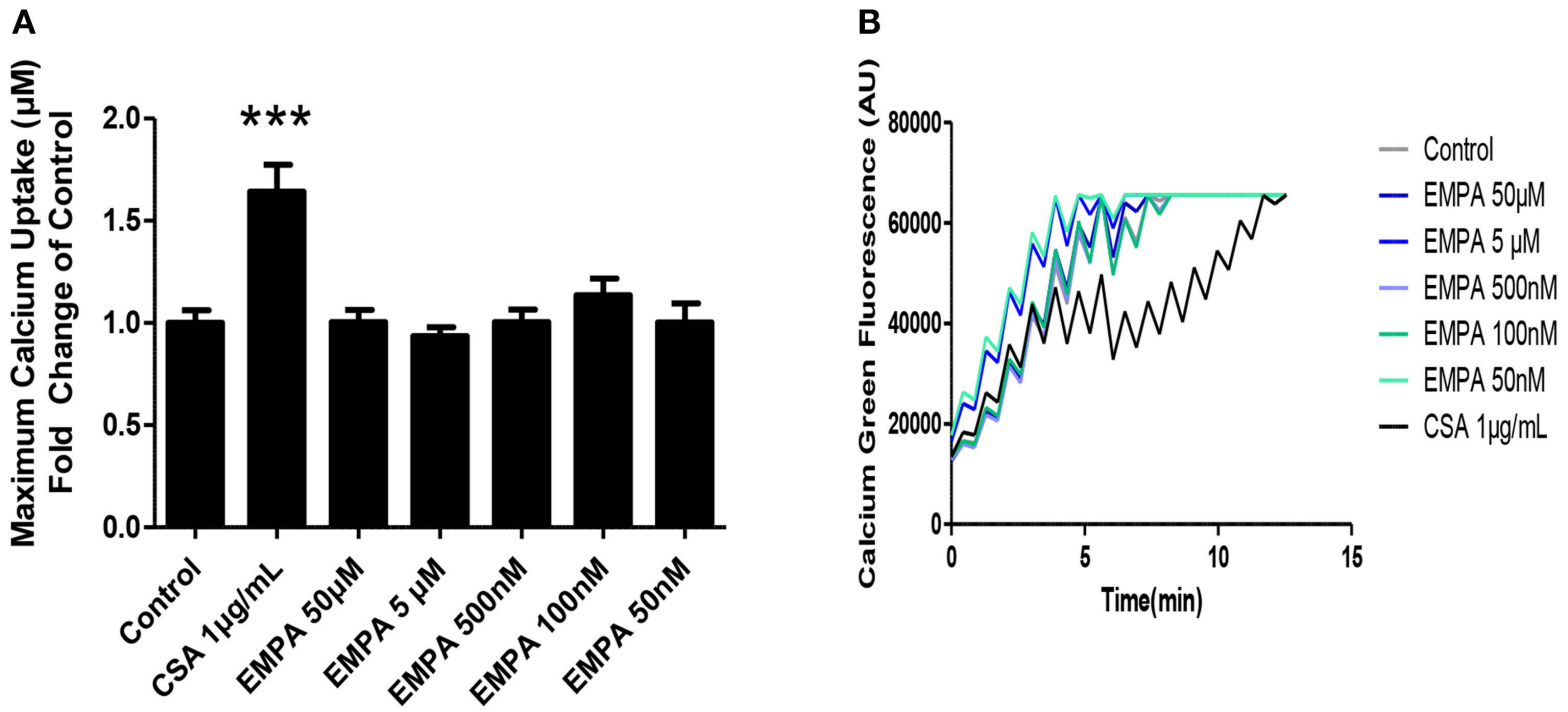
Evaluation of the effects of EMPA on isolated heart mitochondria. Direct effects of empagliflozin on mitochondria. **(A)** Calcium retention capacity of mouse heart mitochondria (^***^p < 0.001 vs. all other study groups) and **(B)** representative Calcium tracing (*n* = 5).

In order to determine the direct effects of EMPA on mitochondrial transition, we recruited an *in vitro* experiment on isolated murine heart mitochondria. EMPA tested in concentrations of 50μM −50 nM had no effect on mitochondrial CRC. Cyclosporine was used as a positive control and increased mitochondrial CRC at a dose of 1 μg/ml (Figures 5A,B; ^***^p < 0.001 vs. all other study groups).

### EMPA protects H9C2 and EC cells against hypoxia/reoxygenation injury in a diabetic milieu

**Figure 6:**
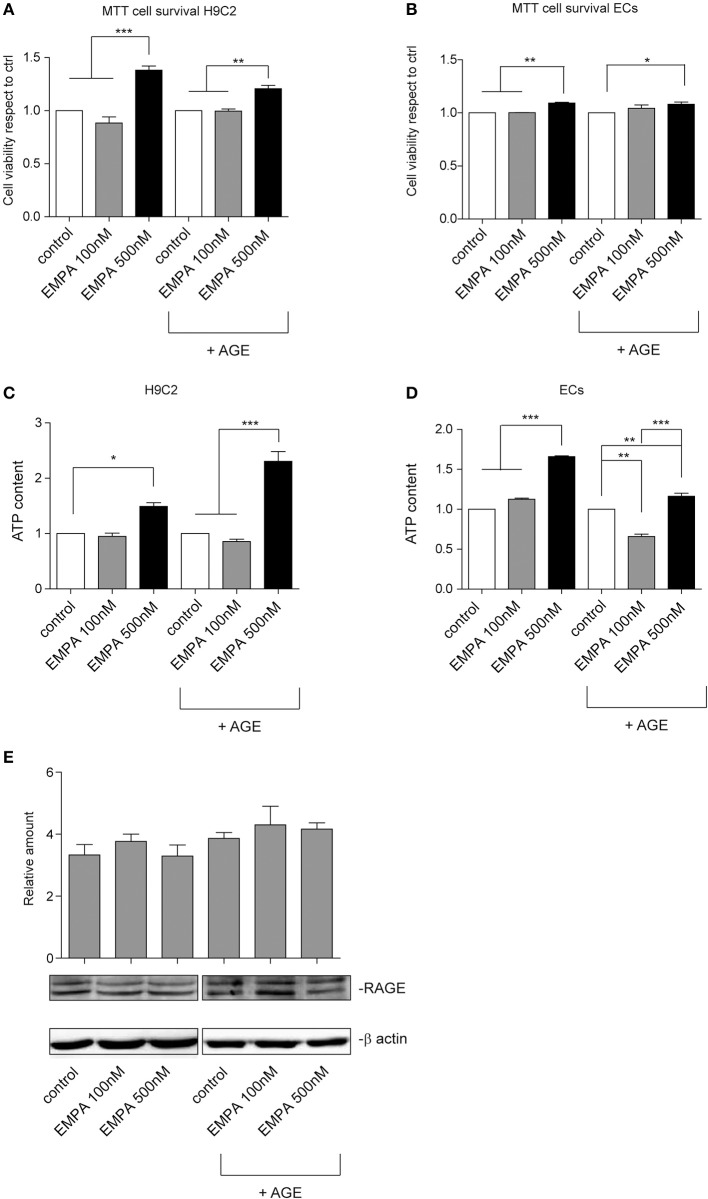
Empagliflozin rescues ECs and H9C2 cells from hypoxia/reoxygenation injury. **(A,B)** MTT assay was used to assess the effect of EMPA in hypoxia/reoxygenation setting. The indicated concentrations were used to treat H9C2 cells **(A)** and ECs **(B)**. Both H9C2 and ECs, either untreated or treated with AGE (1 mg/mL), were subjected to hypoxia/reoxygenation. Data normalized to control are reported as mean ± *SD* and representative of four different experiments performed in triplicate (*n* = 12) (For H9C2, ^***^p < 0.001 EMPA 500 nM vs. control and EMPA 100 nM; ^**^p < 0.01 EMPA 500 nM + AGE vs. control + AGE and EMPA 100 nM + AGE; for ECs, ^**^p < 0.01 EMPA 500 nM vs. control and EMPA 100 nM; ^*^p < 0.05 EMPA 500 nM + AGE vs. control + AGE. **(C,D)** Histogram representation of the relative cellular ATP content. Data are obtained from H9C2 cells and ECs treated as indicated (for H9C2, ^*^p < 0.05 EMPA 500 nM vs. control; ^***^p < 0.001 EMPA 500 nM + AGE vs. control + AGE and EMPA 100 nM + AGE; for ECs, ^***^p < 0.001 EMPA 500 nM vs. control and EMPA 100 nM; ^**^p < 0.01 control + AGE vs. EMPA 100 nM + AGE, EMPA 500 nM + AGE vs. control + AGE, ^***^p < 0.001 EMPA 500 nM + AGE vs. EMPA 100 nM + AGE). Data are reported as mean ± *SD* and representative of four different experiments performed in triplicate (*n* = 12). **(E)** Cell extracts from H9C2 cells treated, with or without AGE (1 mg/mL), and with Empagliflozin at the indicated concentrations, were subjected to hypoxia/reoxygenation and analyzed for RAGE content, and normalized to β-actin Data are representative of four different experiments performed in triplicate (*n* = 12).

In order investigate a direct cardioprotective effect of EMPA we exposed H9C2 and ECs to hypoxia/reoxygenation. EMPA at 500 nM increased H9C2 cell viability and ATP content (Figures 6A,C). More importantly, it was able to protect cells even when AGE was used to mimic the diabetic milieu. Similar results were obtained in ECs (Figures 6B,D). Consistent with AGE expression *in vivo*, no changes in receptor for advanced glycation endproducts (RAGE) expression was detected *in vitro* (Figure 6E).

## Discussion

EMPA treatment in a Type 2 Diabetes animal model, reduced myocardial infarct size, preserved myocardial function after I/R injury, increased cell viability and ATP content in H9C2 and in ECs even when AGE was used to mimic the diabetic milieu. The present study also highlights that EMPA increased STAT3 phosphorylation and expression and reduced myocardial IL-6 and iNOS expressions regulating inflammatory responses and redox signaling in the ischemic myocardium.

Diabetes mellitus increases myocardial susceptibility to I/R injury (Miki et al., 2012). Glycotoxicity plays a major role in this defect by increasing formation of AGE products and the RAGE-induced signaling cascade, leading to vascular dysfunction. RAGE blockade has been demonstrated to be a potential therapeutic approach for treatment of I/R-injury (Park et al., 2015). SGLT2 inhibitors are highly efficient in preventing glucotoxicity, by reducing formation of AGE and induction of RAGE as it is shown in experimental studies (Oelze et al., 2014). In our *in vivo* model EMPA did not alter AGE; additionally, did not alter RAGE expression in H9C2 cells. These findings are consistent with data showing that EMPA, independently of AGE accumulation, exerts pleiotropic protective effects on myocardial function and structure in diabetic db/db mouse (Habibi et al., 2017). In compliance with our findings the administration of EMPA at a similar dose used in our study improved aortic remodeling independently of AGE and RAGE formation in aortic tissue declaring that EMPA activity can be AGE-independent (Oelze et al., 2014).

Reperfusion Injury Salvage Kinase (RISK) and Survivor Activating Factor Enhancement (SAFE) pathways are the main mediators of cardioprotection leading to reduction of infarct size (Heusch, 2015). We did not find any differences between the EMPA and Control groups in the phosphorylation and expression of Akt, eNOS, ERK1/2, and GSK3β in the ischemic myocardium, indicating a RISK independent pathway in EMPA-mediated cardioprotection. This is in agreement with a recent study demonstrating that EMPA did not phosphorylate Akt or ERK1/2 in cardiomyocytes (Habibi et al., 2017). AMPK is a kinase that serves as a key modulator of cellular bioenergetics, and agents acting on AMPK activation, such as metformin, induce cardioprotective effects (Calvert et al., 2008). Canagliflozin, an SGLT2 inhibitor causes a substantial AMPK activation *in vitro*. In contrast, EMPA caused only a modest AMPK activation at high concentrations, indicating that this effect is unlikely to be relevant *in vivo* (Hawley et al., 2016). We confirmed the above findings *in vivo* showing that EMPA did not phosphorylate AMPK, specifying that its cardioprotective effects are independent of AMPK activation. We must mention that all the above signaling events were tested at the time point of the 10th min of reperfusion. This time point was chosen since many of the cardioprotective mechanisms are activated during the first minutes of reperfusion as previously shown (Andreadou et al., 2012, 2015; Kleinbongard et al., 2017). However, components of the RISK pathway such as Akt may be activated after the 10th min of reperfusion (Kleinbongard et al., 2017); therefore we should clarify that in our protocol there is no activation of RISK pathway and AMPK at this specific time point. In addition to the RISK pathway, SAFE and in particular STAT3 activation, is one of the main mediators of triggering cardioprotection (Andreadou et al., 2015; Kleinbongard et al., 2017). Chronic treatment with the SGLT2 inhibitor, dapagliflozin *in vivo*, activated the STAT3 signaling pathway, which on turn enhanced M2 macrophage activation, resulting in the attenuation of cardiac fibrosis molecularly by myocardial iNOS and IL-6 reduced levels (Lee et al., 2017). This effect was more evident with the use of the specific SGLT2 inhibitor dapagliflozin than with the SGLT1/SGLT2 inhibitor phlorizin, implying that compensatory SGLT1 activation after administration of specific SGLT2 inhibitors may play a role in ventricular remodeling (Lee et al., 2017). We found that 6 weeks administration of EMPA resulted in a significant activation of STAT3 at tyrosine 705. We investigated the phosphorylation on Tyr705, since Tyr705 is the primary phosphorylation site of STAT3 (Andreadou et al., 2015). Moreover, the latter study has deduced that STAT3 activation is mediated through decrease of reactive oxygen species (ROS) accumulation in the myocardium. In compliance with the abovementioned findings we have found that in EMPA treated mice circulating levels of MDA are reduced compared to Control. The decrease in lipid peroxidation biomarker MDA seems to be a key element in redox regulating effects of EMPA.

STAT3 phosphorylation has a direct effect on maintaining mitochondrial integrity and attenuating mitochondrial permeability transition pore (MPTP) opening (Heusch, 2015), a key process that is pivotal for the cardiomyocyte survival after ischemia-reperfusion (Bernardi and Di Lisa, 2015; Hausenloy et al., 2016). Additionally, this pore is important in the induction of ROS induced ROS release (RIRR) and can furtherly lead to increased oxidative decay in the cardiomyocytes (Penna et al., 2013). To elucidate whether EMPA exerts its cardioprotective effects through inhibition of MPTP directly, or if the infarct sparing properties are mediated through STAT3 activation, we evaluated the Ca^2+^ retention capacity of isolated heart mitochondria after a series of Ca^2+^ pulses and increasing EMPA concentrations. Since we observed that EMPA did not alter the mitochondrial susceptibility to permeability transition, we concluded that the effect of EMPA in reducing myocardial infarction is STAT3 dependent. However, we must mention that we tested the efficacy of EMPA on mitochondrial transition under normoxic conditions, addressing the direct link between EMPA's cardioprotective potential and mitochondrial retention capacity. Whether the chronic administration of EMPA may alter mitochondrial susceptibility to transition *in vivo*, when subjected to I/R, as well as the underlying induced signaling cascades is a topic of further investigations. Therefore, our next step focused on the signaling cascade complementary to STAT3 activation.

Activation of STAT3, leads to its translocation to the nucleus to function as a transcriptional factor. STAT3 might exhibit a differential role when translocated to the nucleus as it can bind to the promoter of iNOS directly, inhibiting its expression and mitigating cardiomyocyte apoptosis (Su et al., 2016). This is an important finding, as far as the cardioprotective properties of EMPA are concerned, knowing that iNOS is a key contributing molecule in nitrosative stress as well as it possesses deleterious role in inflammatory processes and that diabetes is *per se* a redox-mediated disease. NO derived from iNOS may react with anion superoxide to form peroxynitrite, which may sustain lipid peroxidation (Sag et al., 2013). Therefore, the decreased MDA levels can be directly correlated with the decreased iNOS expression in the EMPA group.

Furthermore, STAT3 can interfere with key molecules implicated in inflammation. NF-κB is a key mediator of inflammatory response, as it leads to the transcription of cytokines and apoptosis-related proteins including IL-6 and iNOS in the myocardium (Gordon et al., 2011). Although we observed reduced levels of expression of both IL-6 and iNOS in the myocardium, we did not find any changes in phosphorylation and/or expression of NF-κB in the EMPA group compared to the Control. Among targets that finely tune NF-κB activity, it is proven that STAT3 acts as a suppressor of NF-κB (Gordon et al., 2011). More specifically, STAT3 can directly interact with NF-κB p65 subunit, leading to a dominant inhibition of NF-κB activity and thus indirectly suppressing cytokine induction of the iNOS promoter independently of NF-κB phosphorylation or expression *in vitro* (Yu et al., 2002). This might be the explanation of our aforementioned findings.

It has been assumed that some of the direct effects of EMPA acting independently of SGLT2 inhibition may be in part responsible for the established cardioprotective effects of the drug. In this aspect a recent study showed that EMPA has direct effects on cardiomyocytes through lowering cytoplasmic sodium [Na^+^]c and calcium [Ca^2+^]c. These effects are mainly mediated via Na^+^/H^+^ exchanger (NHE) activity, independently of SGLT2 (Baartscheer et al., 2017). Therefore, we investigated if EMPA has direct effect in H9C2 cells and in ECs under hypoxia/reoxygenation. EMPA is a very potent and selective SGLT2 inhibitor with an IC50 ~3 nM and Cmax from clinical dosing ~500 nM; concentrations between 100 and 500 nM block effectively and selectively SGLT2 without significant inhibition of SGLT1 (Panchapakesan et al., 2013). Based on the above statements we use 100 and 500 nM and interestingly, we observed that treatment with 500 nM EMPA increased cell viability in comparison to the control in absence or in presence of AGE, and increased ATP content in both cell types. The increased ATP is probably the result of improved mitochondrial function since it has been demonstrated that SGLT2 inhibitors may shift whole-body metabolism from glucose to fat oxidation (Vettor et al., 2017), therefore improving oxidative phosphorylation and mitochondrial respiration.

In a very recent study EMPA reduced pro-inflammatory signaling through amelioration of increased interferon-γ (IFN-γ) in an *in vivo* model of T2D (Steven et al., 2017). From a mechanistic sight of view, EMPA reduced epigenetic changes induced by T2D, as it downregulated the activating epigenetic mark histone3 lysine4 trimethylation (H3K4me3) of the promoters of IFN-γ and iNOS. Moreover, EMPA decreased serum oxidative stress biomarkers namely 3-nitrotyrosine and 4-hydroxynonenal and dose-dependently increased aldehyde dehydrogenase (ALDH-2) activity (Steven et al., 2017), a mitochondrial antioxidant enzyme responsible for the detoxification of tissues from MDA (Wenzel et al., 2007). In compliance with the above-mentioned findings we have shown that EMPA reduced circulating MDA levels and improved mitochondrial function as shown in cells by increased ATP cellular content. Thus, we can speculate that these effects along with the observed reduction of iNOS can be attributed to epigenomic changes present in T2D. Moreover, macrophage infiltration present in the context of AMI and T2D (Lee et al., 2017; Steven et al., 2017) can contribute to the induction of a proinflammatory phenotype and oxidative stress in the myocardium, while SGLT2 inhibition is shown to diminish this process. EMPA's redox regulating effects seem to be pleiotropic as it acts both by upregulating endogenous antioxidant mechanisms and interfering with epigenomic changes associated with T2D.

Conclusively, EMPA reduces myocardial infarct size in animals fed with WD through STAT3 activation and regulation of inflammatory responses in the myocardium. Moreover, the decrease in iNOS expression and the concomitant decrease in lipid peroxidation is of great importance. While diabetes is a redox disease, the redox regulation by EMPA in parallel with its glucose-lowering effects can be pivotal in managing diabetes and limiting myocardial infarction. Therefore, the assessment of EMPA effects on myocardial necrosis and the elucidation of the molecular mechanisms responsible for its cardioprotection are of paramount importance. It would allow predicting whether EMPA has such beneficial effects also in diabetic patients without prior cardiovascular disease, or in non-diabetic patients with cardiovascular disease.

## Study limitations

We know that SGLT2 is highly specifically expressed in the kidney and very minimally in the heart (Chen et al., 2017). Therefore, additional studies are essential in order to investigate how EMPA would exert its effects on cardiomyocytes. One of the main limitations of the present study is the mechanistic insight of the role of STAT3 mediating cardioprotection. Additional studies to elucidate the exact cardioprotective mechanism of EMPA by the use of established inhibitors *in vivo* are necessary to answer this question. However, our study is able to stimulate research in investigating the cardioprotective mechanisms of this drug in the setting of I/R injury.

## Author contributions

IA, MB, GD, and EI: contributed to conception and design, contributed to acquisition, analysis, and interpretation, drafted the article, critically revised the article and gave final approval; PE, EB, GT, CHD, AV, P-EN, CAD, EM, VL, II, and NK: contributed to acquisition, analysis, and interpretation, critically revised the article, and gave final approval.

### Conflict of interest statement

The authors declare that the research was conducted in the absence of any commercial or financial relationships that could be construed as a potential conflict of interest.
