# Supplementary material for: Empagliflozin Limits Myocardial Infarction in Vivo and Cell Death in Vitro: Role of STAT3, Mitochondria, and Redox Aspects
Source: Front Physiol. 2017 Dec 19;8:1077. doi: 10.3389/fphys.2017.01077 (PMC5742117; doi:10.3389/fphys.2017.01077)
Supplement: Supplementary file 1 [file Table1.DOC]

**Supplementary Data**

**Supplementary Materials and Methods**

**Supplementary Figures**

**Empagliflozin limits myocardial infarction in vivo and cell death in vitro: role of STAT3, mitochondria, and redox aspects.**

Ioanna Andreadou^1a*^, Panagiotis Efentakis^1a^, Evangelos Balafas^2^, Gabriele Togliatto^3^, Constantinos H. Davos^4^, Aimilia Varela^4^, Constantinos A. Dimitriou^4^, Panagiota-Efstathia Nikolaou^1^, Eirini Maratou^5^, Vaia Lambadiari^6^, Ignatios Ikonomidis^7^, Nikolaos Kostomitsopoulos^2^, Maria F. Brizzi^3^, George Dimitriadis^6^, Efstathios K. Iliodromitis^7^

*^1^National and Kapodistrian University of Athens, Laboratory of Pharmacology, Faculty of Pharmacy, Athens, Greece; ^2^Academy of Athens Biomedical Research Foundation, Centre of Clinical Experimental Surgery and Translational Research, Athens, Greece; ^3^Department of Medical Sciences, University of Turin, Corso Dogliotti 14, 10126, Turin, Italy; ^4^Cardiovascular Research Laboratory, Biomedical Research Foundation, Academy of Athens, Greece; ^5^Hellenic National Center for Research, Prevention and Treatment of Diabetes Mellitus and its Complications, Athens, Greece; ^6^2nd Department of Internal Medicine, Research Institute and Diabetes Center, National and Kapodistrian University of Athens, "Attikon" University Hospital, Athens, Greece; ^7^2nd University Department of Cardiology National and Kapodistrian University of Athens, Medical School, Athens, Greece*

**Corresponding author:** Ioanna Andreadou, PhD, Faculty of Pharmacy, National and Kapodistrian University of Athens, Panepistimiopolis, Zografou, Athens 15771, GREECE; tel:+30 210 7274827; fax:+30 210 7274747; e-mail: [jandread@pharm.uoa.gr](mailto:jandread@pharm.uoa.gr)

^a^Equal contributed authors

**Supplementary Methods**

**Empagliflozin isolation**

Ten commercially available film-coated tablets (Jardiance F.C. Tab 25mg®, Boehringer Ingelheim, Germany) were pulverized and the powder was suspended in absolute Ethanol 99%. The formed suspension was filtered through a pleated filter and the filtrate was centrifuged at 4000xg for 10 minutes twice for the removal of the insoluble excipients. The solution was then vacuum vaporized and empagliflozin was received as a crystal weight powder. The purity of the compound was verified via ^1^H-NMR spectroscopy NMR spectrum was compared to the literature and found identical (ApexBio Technology, USA). ^1^H-NMR spectra is shown in Figure S1.

**Supplementary Figure Legends**

**Figure S1**: 1D-^1^H-NMR spectrum of empagliflozin in DMSO at 600 MHz

**Figure S2**: M-mode echocardiography of control and EMPA groups at baseline and 14 weeks. Left ventricular end-diastolic (EDD) and end-systolic dimensions (ESD) are improved in EMPA group compared to the Control group.


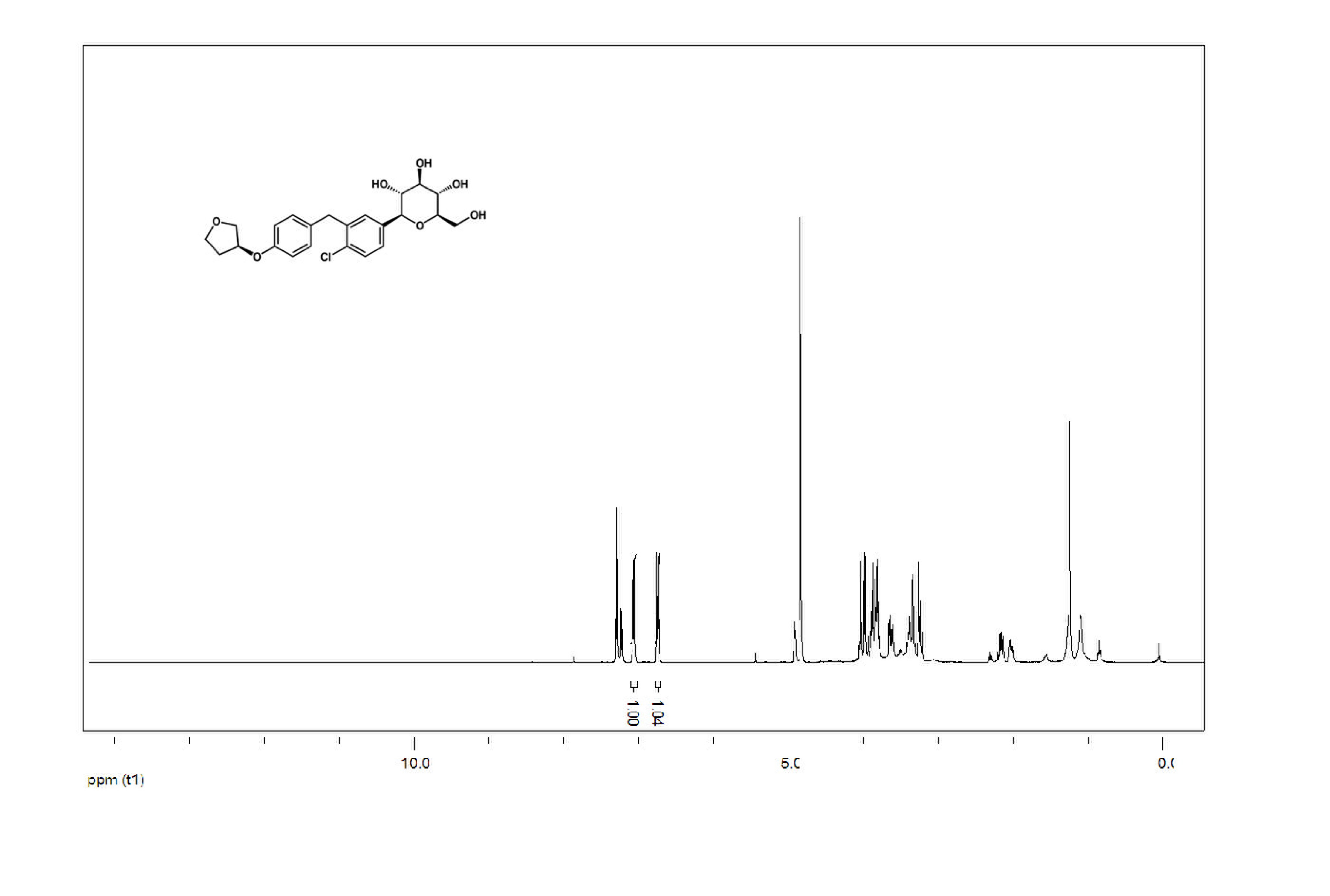


Figure S1


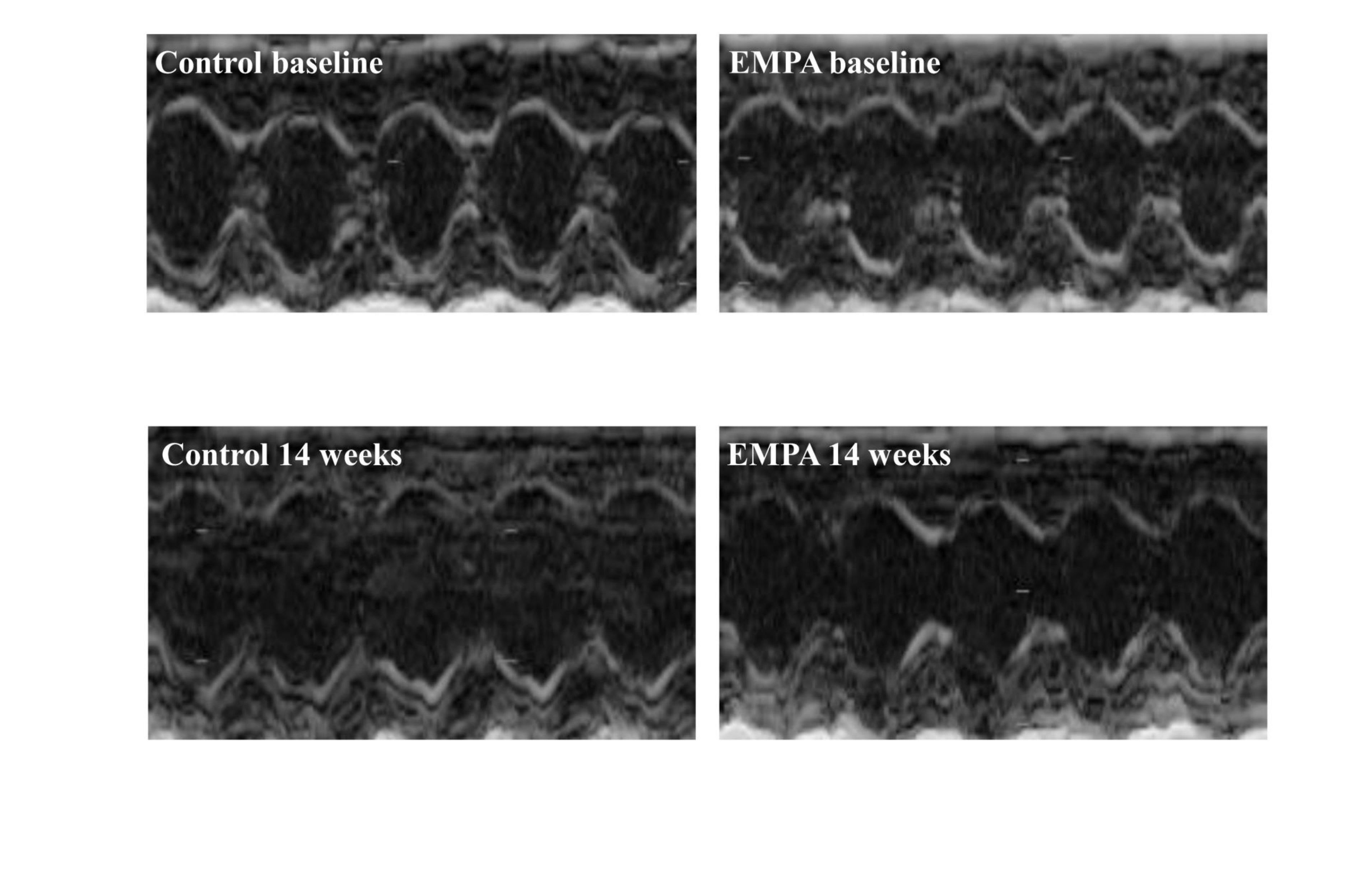


Figure S2
